# The Lassa Virus Stable Signal Peptide Undergoes a Conformational Change to Aid Viral Fusion

**DOI:** 10.1002/chem.202403608

**Published:** 2025-03-07

**Authors:** Shane D. Collins, Liqun Jiang, Yanxin Liu, Jinwoo Lee

**Affiliations:** ^1^ Department of Chemistry and Biochemistry College of Computer, Mathematics, and Natural Science University of Maryland College Park MD, 20740 USA; ^2^ Institute for Bioscience and Biotechnology Research University of Maryland Rockville MD 20850 USA

**Keywords:** Membrane Proteins, Membrane Fusion, Viruses, NMR Spectroscopy, Fluorescence

## Abstract

A critical event in the lifecycle of the Lassa virus (LASV) is membrane fusion, where the viral membrane merges with the host cell membrane. This process is initiated by the LASV surface glycoprotein complex (GPC) upon exposure to the acidic environment of the endocytic pathway. A unique aspect of the GPC is the stable signal peptide (SSP), located adjacent to the transmembrane region of glycoprotein 2 (GP2), the primary fusion subunit. While previous research has established the importance of SSP in fusion, its precise role remains to be determined due to limited biophysical data. Our study aims to elucidate SSP's role by examining its structural changes. We discovered that SSP is predominantly α‐helical in its prefusion state at pH 7. However, when the pH is lowered to mimic the late endosomal environment (<pH 5), SSP undergoes a structural change, increasing its helical content. Moreover, we demonstrate that SSP directly enhances fusion at these acidic pH levels. In conclusion, our findings suggest that SSP undergoes a critical conformational change at low pH, which is essential for its role in the LASV fusion mechanism, thereby deepening our understanding of LASV fusion.

## Graphical abstract

The unique stable signal peptide (SSP) of the Lassa virus glycoprotein complex undergoes a conformational change during pH‐dependent viral fusion. This revels a potential mechanism by which the enigmatic SSP influences the fusion process through interactions it makes with the fusion protein, glycoprotein 2 (GP2).

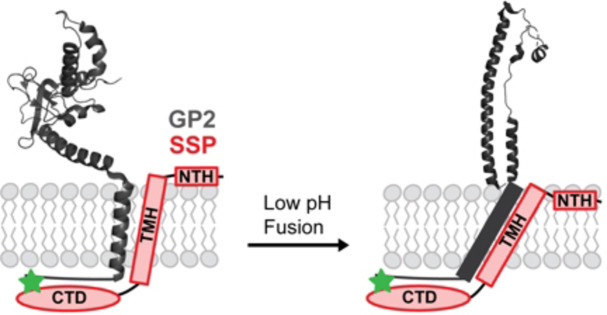

## Introduction

**Figure 1:**
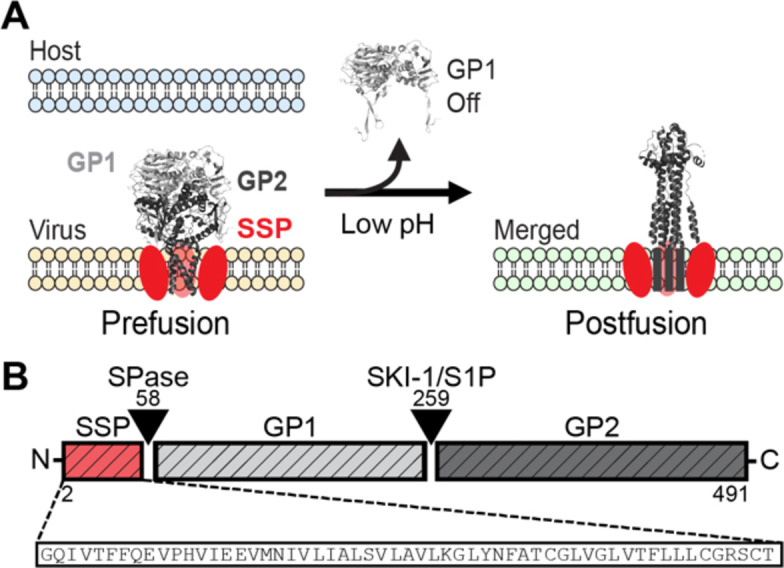
(A) The LASV GPC exists in its prefusion state as the virus encounters a host membrane. Low pH in a late endosome/lysosome (~pH 5‐4) and a receptor switch event (not shown) leads to dissociation of GP1 and subsequent GP2 mediated fusion of the host and viral membranes. SSP, which interacts with GP2, is a unique and required component of the LASV fusion mechanism. GPC structures taken from PDBs 7PUY and 5OMI. (B) LASV GPC is expressed as a single polypeptide. Cleavage at two sites leads to the production of the SSP, GP1, and GP2 subunits that form the mature GPC. SSP sequence is shown below.

Lassa virus (LASV) is the prototypical species of the arenavirus family and is endemic to West Africa where it has been a significant public health burden for many years.[^1^, ^2^] The World Health Organization designated LASV as a priority research target due to its current public health burden and potential for causing a future pandemic.^[3]^ As an enveloped virus, LASV has evolved specific machinery to carry out the complex series of steps required to infect a host cell. This is accomplished by the glycoprotein complex (GPC), a trimer of trimers comprised of: the receptor binding subunit, glycoprotein 1 (GP1); membrane fusion subunit, glycoprotein 2 (GP2); and the uniquely retained stable signal peptide (SSP).[^4^, ^5^, ^6^] LASV virions enter the host cell after GP1 engages an α‐dystroglycan receptor on the cell surface.[^7^, ^8^, ^9^] Subsequently, the virion is endocytosed and delivered late within the endocytic pathway where the acidic environment causes GP1 to undergo a receptor switch to LAMP1 (lysosomal associated membrane protein 1). This triggers GP1 dissociation, leading to conformational changes within GP2 and subsequent fusion initiation.[^10^, ^11^, ^12^] LASV GP2 and its fusion mechanism are generally analogous to that of other class I fusion proteins, which also includes the fusion machinery of other viruses such as influenza, Ebola, and HIV (Human Immunodeficiency virus).[^13^, ^14^, ^15^, ^16^] Collectively, this mechanism is referred to as the six‐helix bundle fusion mechanism due to the close‐fitting interaction of six helices in the postfusion structure of the fusion protein. Interestingly, SSP is a required component of the LASV fusion process, adding a unique piece to the LASV fusion machinery and mechanism (Figure 1A).[^17^, ^18^] However, the role of SSP within the fusion process remains largely unknown.

The SSP is expressed at the N‐terminus of the GPC polypeptide and is cleaved by cellular SPase (signal peptidase) to generate a peptide that is 58 amino acids in length (Figure 1B).^[19]^ SSP performs traditional signaling peptide functions for the LASV glycoprotein including targeting the nascent GPC to the ER membrane, facilitating transport of the GPC through the endoplasmic reticulum‐Golgi pathway to the host plasma membrane, and enabling enzymatic maturation of the GPC into its active components.[^19^, ^20^, ^21^] Notably, SSP has several unique aspects, such as being much larger than traditional signal peptides, which are typically ~20 amino acids.^[22]^ Furthermore, SSP is not degraded after completing its traditional signal peptide functions, rather SSP remains a part of the GPC within the mature virion. SSP's size and localization near the LASV fusion machinery, specifically GP2, likely contributes to its extraordinary role in the LASV fusion mechanism.[^19^, ^23^]

The SSP is hypothesized to have three distinct regions: an N‐terminal helix (NTH), a transmembrane helix (TMH), and a C‐terminal domain (CTD). Recently, a cryo‐EM structure of LASV GPC unveiled a portion of SSP, providing insight into its prefusion state.^[9]^ This structure captured the NTH and TMH, highlighting their contribution, although CTD was not present in the structure due to resolution limitations.^[9]^ Previous cell‐based studies involving LASV and other related arenaviruses have shown point mutations of SSP alter fusion efficiency drastically, highlighting the importance of SSP in the LASV fusion mechanism.[^18^, ^24^, ^25^] Studies using chimeras of SSP have indicated that the NTH and/or TMH are likely responsible for SSP function within the fusion process, meanwhile the CTD likely has no direct role in fusion.^[17]^ Further studies have proposed that specific SSP interactions with GP2 impact LASV fusion. Mutational studies showed SSP likely forms an interface with the transmembrane domain (TM) of GP2, which was later confirmed by the cryo‐EM structure of GPC.[^9^, ^17^, ^26^, ^27^] Additionally, multiple mutagenesis studies using small molecular fusion inhibitors have found their binding site to be at the GP2‐SSP interface, likely between the TM of GP2 and TMH of SSP.[^28^, ^29^, ^30^, ^31^, ^32^] These inhibitors were found to lower cell‐to‐cell fusion, suggesting the molecules disrupted GP2‐SSP interactions, leading to perturbation of fusion. Meanwhile, the CTD of SSP is thought to form a zinc‐binding complex with the cytoplasmic tail of GP2 that may add binding fidelity between the two subunits; however, this interaction likely has no direct role during fusion.[^17^, ^33^, ^34^] Taken together, SSP's role within fusion is related to its interactions with GP2. Despite these efforts, the mechanism of how GP2‐SSP interactions impact fusion remains unclear, mainly due to a lack of biophysical knowledge of SSP.

This study aims to address the role of SSP in LASV fusion by better characterizing the structure of SSP, including how SSP structure is affected by a pH that mimics the fusion environment of LASV. We first designed a protocol to incorporate SSP into both detergent micelles and phospholipid liposomes, which enables various methods of *in vitro* analysis. Through circular dichroism (CD) and nuclear magnetic resonance (NMR), we found SSP's prefusion state (pH 7) is mostly helical, whereas at a low pH that resembles a late endosome/lysosome (<pH 5) SSP underwent a conformational change that corresponds with an increase in helical content. Furthermore, tryptophan (Trp) fluorescence experiments show that the NTH and TMH of SSP are the primary locations undergoing a conformational change at low pH. Finally, we demonstrate with an *in vitro* fusion assay that the presence of SSP directly increases LASV fusion at the pH of a late endosome/lysosome. Taken all together, we believe the low pH conformational change undergone by SSP is crucial towards the LASV fusion mechanism.

## Materials and Methods

### Expression and Purification

A LASV SSP construct containing an N‐terminal His tag, Trp leader tag to aid with expression, and thrombin cleavage site was designed and inserted into a pET24 vector by Genscript.^[35]^ Notably, the non‐conserved C41 and C53 residues were mutated to simplify purification based on sequence conservation of other arenaviruses (Figure S1). The plasmid was transformed into BL21(DE3) pLysS *Escherichia coli* cells (New England Biolabs). A single colony was used to inoculate 2x YT media with appropriate antibiotics, which was then incubated at 37 °C until an optical density of 0.6–0.8 was reached. The cells were induced with a final concentration of 1 mM IPTG (Isopropyl β‐D‐1‐thiogalactopyranoside) and incubated for 4 hrs at 37 °C until harvesting. After harvesting, 0.5 L of cells were solubilized in 120 mL sucrose buffer (20 % sucrose, 300 mM NaCl, 20 mM Tris, 10 mM β‐mercaptoethanol (BME), pH 8.0) and sonicated on ice for 10 min (1 s on/1 s off cycles, 40 % amplitude) with a probe sonicator. The solution was spun down at 40000xg for 1 hr at 4 °C, whereupon the resulting pellet was resuspended in 50 mL binding buffer (8 M urea, 1 % Triton X‐100, 40 mM imidazole, 300 mM NaCl, 20 mM tris, 10 mM BME, pH 8.0) and sonicated with the same parameters as above. The solution was spun down at 40000xg for 1 hr at 4 °C and the supernatant was added to ~5 mL rinsed Ni‐NTA resin. After overnight incubation, the flow through was collected from the column and the resin was washed with 100 mL binding buffer, then 50 mL wash A buffer (8 M urea, 125 mM imidazole, 300 mM NaCl, 20 mM tris, 10 mM BME, pH 8.0), and finally 100 mL wash B (300 mM NaCl, 20 mM tris, pH 8.0). 15 mL cleavage buffer (30 mM βOG (n‐octyl‐β‐D‐octylglucoside), 100 mM NaCl, 20 mM tris, 2.5 mM CaCl_2_, pH 8.5) was then added to the column with 33 μ g/mL thrombin and allowed to incubate overnight at room temperature. The cleavage buffer was flowed out and the column was rinsed with 100 mL wash B buffer, 60 mL wash C buffer (8 M urea, 250 mM imidazole, 300 mM NaCl, 20 mM tris, 10 mM BME, pH 8.0), and finally 100 mL wash B buffer. 10 mL of either TCA elution buffer (4.5 M TCA (trichloroacetic acid), 300 mM NaCl, 20 mM Tris, pH 8.0) or LMPG elution buffer (2 mM LMPG (1‐myristoyl‐2‐hydroxy‐sn‐glycero‐3‐phospho‐[1′‐rac‐glycerol] (sodium salt)) (Avanti Polar Lipids), 100 mM NaCl, 25 mM Na_2_HPO_4_, pH 7.0) was added and allowed to incubate overnight at room temperature or 4 °C, respectively. The elutions were collected and stored until required. A representative SDS‐PAGE gel is shown in Figure S2B. LASV GP2 was expressed and purified from a previously established protocol.^[36]^

### Incorporation into Liposomes

Appropriate amounts of POPC (1‐palmitoyl‐2‐oleoyl‐glycero‐3‐phosphocholine) and POPG (1‐palmitoyl‐2‐oleoyl‐sn‐glycero‐3‐phospho‐[1′‐rac‐glycerol]) (Avanti Polar Lipids) in chloroform were combined in a glass test tube and dried down with gentle vortexing under a stream of N_2_ and placed in a vacuum desiccator overnight. Samples were prepared by adding SSP in TCA buffer to the tube containing the lipids. The TCA concentration was diluted to 3 M and βOG and 1 mM TCEP (tris(2‐carboxyethyl)phosphine) were added to the solution and the lipids were then resuspended via vortexing. The final volume was 500 μ L. The ratios of the mixture were dependent on the experiment; 10000 : 2000 : 1 β‐OG : lipid : protein was used for DLS (dynamic light scattering), cryo‐EM, and lipid mixing, while 2500 : 500 : 1 was used for CD (circular dichroism) and fluorescence. Every experiment used a 75 : 25 POPC : POPG composition. When samples required GP2, GP2 in TCA buffer was added to the mixture before resuspending the lipids at a 1 : 1 GP2 : SSP ratio. After placing on a room‐temperature shaker for ~1 hr, the mixture was passed through a Sephadex^TM^ G‐25 fine resin column with an internal diameter of 10 mm and a bed height of 250 mm. 0.5 mL fractions were collected and fractions containing the proteoliposomes were confirmed via nanodrop, pooled, and diluted to 2 mL.

### Dynamic Light Scattering

Liposomes containing GP2‐SSP were made to 62.5 nM SSP, 62.5 nM GP2, 125  μM lipids, 10 mM HMA (HEPES, MES, Sodium Acetate), 100 mM NaCl, pH 7. SSP alone liposomes had the same composition but without GP2. Samples were run on a nanoPARTICA Nanoparticle Analyzer SZ‐100V2 (Horiba Scientific) in a plastic cuvette with a volume of 750uL at room temperature. Six trials were run to acquire an average mean particle size.

### Cryo‐EM Sample Preparation and Data Acquisition

Liposomes containing GP2‐SSP were prepared to 125 nm GP2, 125 nm SSP, 250 μM lipids, 10 mM HMA, 100 mM NaCl, pH 7. Cryo‐EM grids were prepared with Vitrobot Mark IV (ThermoFischer Scientific) at 10 °C and 100 % humidity. 4 μL aliquots of samples were applied to glow discharged Quantifoil R1.2/1.3, 300‐mesh copper holey carbon grids (Quantifoil Micro Tools), single blotted for 10 seconds with blot force 0, and plunge frozen in liquid ethane cooled by liquid nitrogen. Cryo‐EM images of the proteoliposome were acquired on 200 kV Glacios 2 (ThermoFischer Scientific) equipped with a Falcon IV camera. Images were recorded with EPU with a pixel size of 0.92 Å. Each image was fractionated to 1440 frames with a total dose of 34 e/Å^2^.

### Circular Dichroism

All samples were run on a J810 Spectro‐Polarimeter (JASCO) using a quartz cuvette with a 2 mm path length. SSP in LMPG micelles were made to 20 μM protein, 10 mM LMPG, 1 mM HMA, 10 mM NaCl, pH 7 while SSP in liposome were made to 4 μM protein, 2 mM lipids, 1 mM HMA, 10 mM NaCl, pH 7. The pH of the sample was dropped to the appropriate pH using 1 M HCl and the pH was confirmed with a micro pH probe. The spectra were acquired at 100 nm/min scanning speed and averaged over 12 acquisitions. Blanks of the appropriate solution and pH conditions were subtracted from the sample data. Data was collected at room temperature. Spectra were then processed using the program CDToolX.^[37]^ The spectra were converted from millidegrees to MRE (mean residue ellipticity) and the helical percentage, *f*_H_, was calculated from the MRE at 222 nm via the following equation: *f*_H_=(θ_222_−θ_C_)/(θ_H_−θ_C_) where θ_C_=2220−53 *T*, θ_H_=(250 *T*−44000)(1−3/*n*), *T* is the temperature in Celsius, and *n* is the number of residues in the protein.^[38]^

### Nuclear Magnetic Resonance

To make ^15^N‐isotopically labeled SSP, the same expression and purification protocol was used with modifications to improve expression and isotopic incorporation.^[41]^ Cells were first grown in 4 L of LB until an OD of 0.4–0.5 was reached, at this point, the cells were spun down at 4000xg at 15 °C for 10 min. The cells were resuspended in minimal media without carbon or nitrogen sources (8 g/L Na_2_HPO_4_, 2 g/L KH_2_PO_4_, 0.5 g/L NaCl, 1 mM MgSO_4_, 0.3 mM Na_2_SO_4_, 0.3 mM CaCl_2_, pH 7.2) and then spun down again with the above centrifuge parameters to pellet out the cells. This step removes residual LB from the cells to maximize isotopic labeling. The cells were then combined and resuspended in 1 L ^15^N minimal media (8 g/L Na_2_HPO_4_, 2 g/L KH_2_PO_4_, 0.5 g/L NaCl, 10 g/L dextrose, 1 g/L ^15^N NH_4_Cl (Cambridge Isotopes), 1 mM MgSO_4_, 0.3 mM Na_2_SO_4_, 0.3 mM CaCl_2_, 100 mg/L biotin, 100 mg/L thiamine, pH 7.2) and allowed to shake at 37 °C for 1 hr to acclimate the cells. The cells were then induced with a final concentration of 1 mM IPTG and grown for 4 hrs at 37 °C until harvesting. NMR samples were run on an Ascend 800 MHz magnet (Bruker) equipped with a CPQCI cryoprobe. Sample acquisition was done at 37 °C. Samples were made to ~250 μM SSP, 250 mM LMPG, 25 mM Na_2_HPO_4_, 100 mM NaCl, pH 7. Sample pH was dropped using 1 M HCl and confirmed using a micro pH probe. Data was processed using NMRPipe and NMRFAM‐SPARKY via NMRBox.[^42^, ^43^, ^44^]

### Tryptophan Fluorescence

Site‐directed mutagenesis PCR (polymerase chain reaction) was performed using Q5 polymerase (New England Biolabs) to generate each single point Trp mutant. Expression and purification of the SSP Trp mutants was the same as for wild‐type SSP. Mutant samples were made to 125 nM SSP, 250 μM lipids, 10 mM HMA, 100 mM NaCl, pH 7. Concentrations were kept below OD>0.1 at all excitation wavelengths to avoid inner filter effects.[^45^, ^46^] For steady state fluorescence emission spectra, the excitation wavelength was set to 275–290 nm, while the emission window was set to 310–400 nm. All fluorescence data was obtained with a Fluorolog‐3 Max (Horiba Scientific) at room temperature with emission and excitation bandpass set to 5 nm each. A square cuvette with a 2 mm pathlength was used. A liposome blank at the corresponding pH was subtracted from each spectrum to correct for scattering due to the large size of the liposomes.

### Lipid Mixing Assay

The lipid mixing samples were made similar to the other liposome preparations with one alteration; 1 mM ZnCl_2_ was added to the sample before being passed through the Sephadex^TM^ G‐25 fine resin column. Viral mimic liposomes containing GP2‐SSP were made to 125 nm SSP, 125 nm GP2, 250 μM lipids, 10 mM HMA, 100 mM NaCl, pH 7. GP2 only and SSP only liposomes had the same composition but without SSP and GP2, respectively. Host mimic liposomes were produced by an extrusion method briefly noted here. An appropriate amount of POPC, POPG, RH‐PE (1,2‐dioleoyl‐sn‐glycero‐3‐phosphoethanolamine‐N‐[lissamine rhodamine B sulfonyl]), and NBD‐PE (1,2‐dioleoyl‐sn‐glycero‐3‐phosphoethanolamine‐N‐[7‐nitro‐2–1,3‐benzoxadiazol‐4‐yl]) (Avanti Polar Lipids) were added to a glass test tube and dried down with gentle vortexing under a stream of N_2_ stream and placed in a vacuum desiccator overnight. The lipids were resuspended in 500 μ L 10 mM HMA, 100 mM NaCl, pH 7. The lipids were subjected to 10× freeze/thaw cycles in a liquid N^2^/42 °C water bath. Finally, the lipids were passed through the Liposofast extrusion kit (Avestin) containing two 100 nm pore size polycarbonate membranes 21 times. The final concentration was 1 mM lipids with a composition of 73 : 25 : 1 : 1 POPC : POPG : RH‐PE : NBD‐PE.

54 μ L of viral mimic liposomes, 1.5μL of host mimic liposomes, and 94.5μL of HMA buffer were combined in a well of a black walled/clear bottom plate. All data was collected on a SpectraMax M5 plate reader (Molecular Devices) at room temperature. Excitation and emission wavelengths were set to 460 nm and 538 nm, respectively, while a 530 nm filter was used. After collecting a baseline reading for each well, the pH was dropped using an appropriate amount of 1 M HCl, and the fluorescence reading was taken until the reading leveled off. Finally, a fluorescence reading was taken after adding 6.5 μ L of 25 % Triton X‐100 to the well to burst all the liposomes. To calculate %Fusion, the following formula was used: %Fusion=(F_H_−F_B_)/(F_T_−F_B_), where F_B_ is the baseline fluorescence after mixing the host and viral membrane mimics at pH 7, F_H_ is fluorescence after dropping the pH with 1 M HCl, and F_T_ is fluorescence after adding Triton X‐100. %Fusion of blank liposomes was subtracted at each corresponding pH.

### Content Mixing

Content mixing was performed similarly to lipid mixing with slight modifications. Before resuspending the labeled lipids containing RH‐PE and NBD‐PE with HMA buffer, 3.125 mM ANTS and 11.25 mM DPX, both soluble dyes, were added to the tube. After resuspension, freeze/thawing, and extruding, the lipids were passed through a PD‐10 desalting column (Cytiva Life Sciences) to remove ANTS and DPX not encapsulated by the liposomes. Fractions containing the labeled liposomes were pooled and added to the wells while adjusting for dilution caused by the column. 30 mM DPX was added to the wells to eliminate signal from ANTS caused by liposome bursting. Lipid mixing and content mixing were run concurrently by exciting at 460 nm and 355 nm and reading emission at 538 nm and 520 nm, respectively. 530 nm and 515 nm filters were used, respectively.

## Results and Discussion

### Purification of LASV SSP into Different Membrane Mimetic Systems

**Figure 2:**
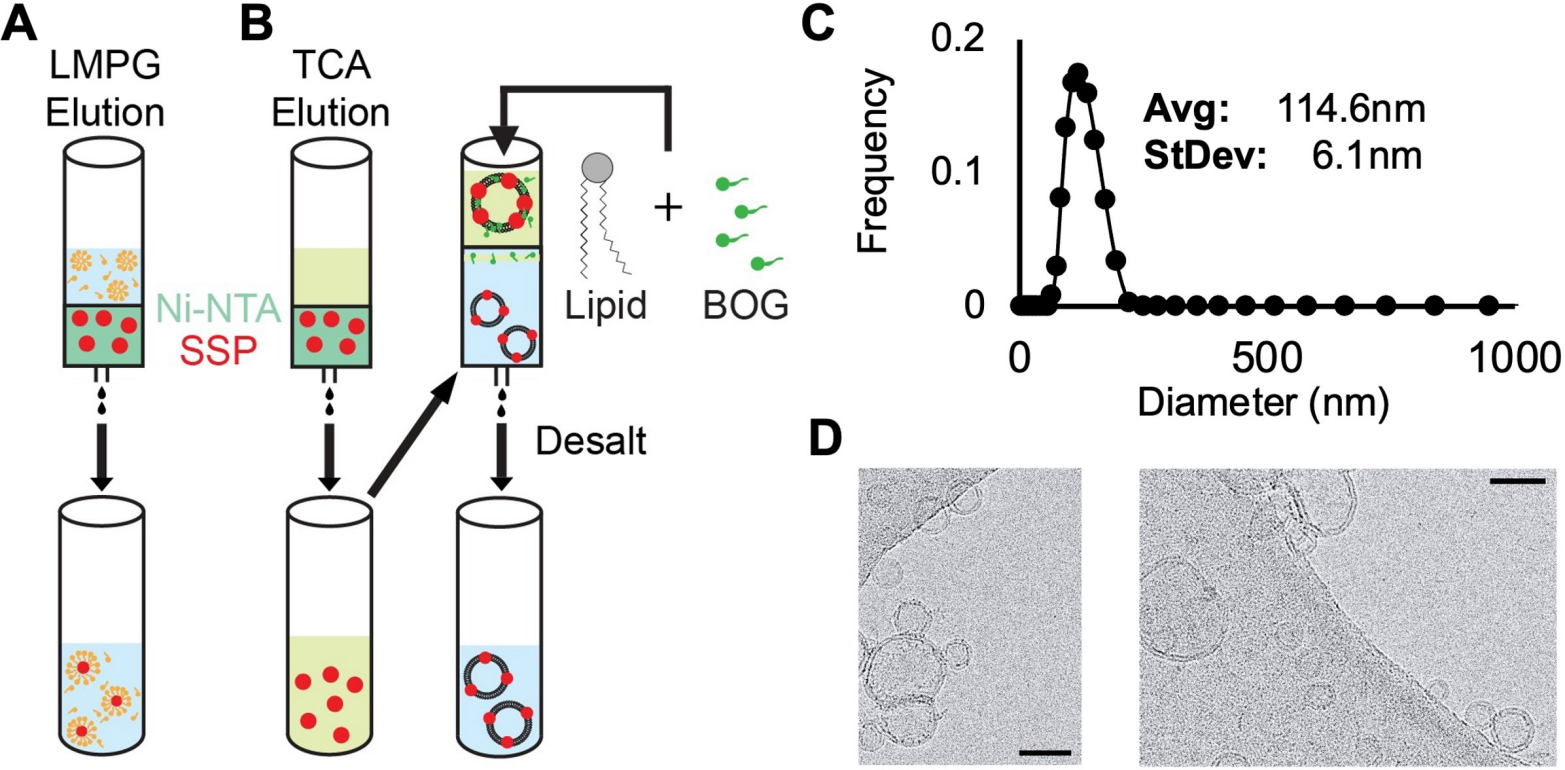
Illustration of LASV SSP incorporation into (A) LMPG micelles and (B) phospholipid liposomes. Refer to Materials and Methods section for further detail on liposome incorporation. (C) Diameter distribution of SSP incorporated liposomes acquired with DLS. (D) Cyro‐EM images of GP2‐SSP proteoliposomes indicated by arrows (scale bars=50 nm). Liposomes produced from this method are confirmed to be unilamellar with uniform size. Lipid compositions were 75 : 25 POPC : POPG.

We designed a purification system to facilitate the incorporation of isolated SSP into various membrane mimetic systems. This system utilizes an expression construct featuring an N‐terminal His‐tag and thrombin cleavage site, enabling a streamlined purification protocol that will result in purified SSP without any additional residues or tags (Figure S2A). Following a purification scheme similar to that previously established for other LASV membrane proteins, SSP was successfully incorporated into either LMPG micelles or phospholipid liposomes (Figure 2A–B).^[47]^

While our method of generating proteoliposomes resembles previously established protocols utilizing detergent‐assisted incorporation, there are some key differences, mainly the presence of the denaturant TCA in the protein‐lipid‐detergent solution before passing through a gel filtration column.[^48^, ^49^, ^50^] To assess the quality of liposomes produced by this modified protocol, we performed DLS analysis. The results show that liposomes containing SSP exhibited an average diameter of 114.6 nm and displayed a normal distribution with a standard deviation of 6.1 nm (Figure 2C). Notably, the average size of liposomes comprised of SSP alone was not significantly different from liposomes containing GP2‐SSP complex (Figure S3). In addition, Cryo‐EM imaging confirmed that the liposomes produced were unilamellar, which is crucial in ensuring the liposomes model biological membranes (Figure 2D). Taken together, these results demonstrate that our protocol reliably produces unilamellar liposomes of uniform size containing LASV SSP and GP2, making them suitable for further structural and functional analysis. Furthermore, this method is effective for incorporating both small (SSP = ~6 kDa) and medium (GP2 = ~25 kDa) sized proteins into liposomes. Therefore, we believe it can be expanded to be used with other challenging membrane proteins for *in vitro* biophysical characterization. This includes proteins, such as those studied here, that are insoluble in ‘weak’ detergents with high critical micelle concentrations, which are commonly used in traditionally detergent‐assisted membrane incorporation.

### Structural Change Undergone by LASV SSP at Low pH

**Figure 3:**
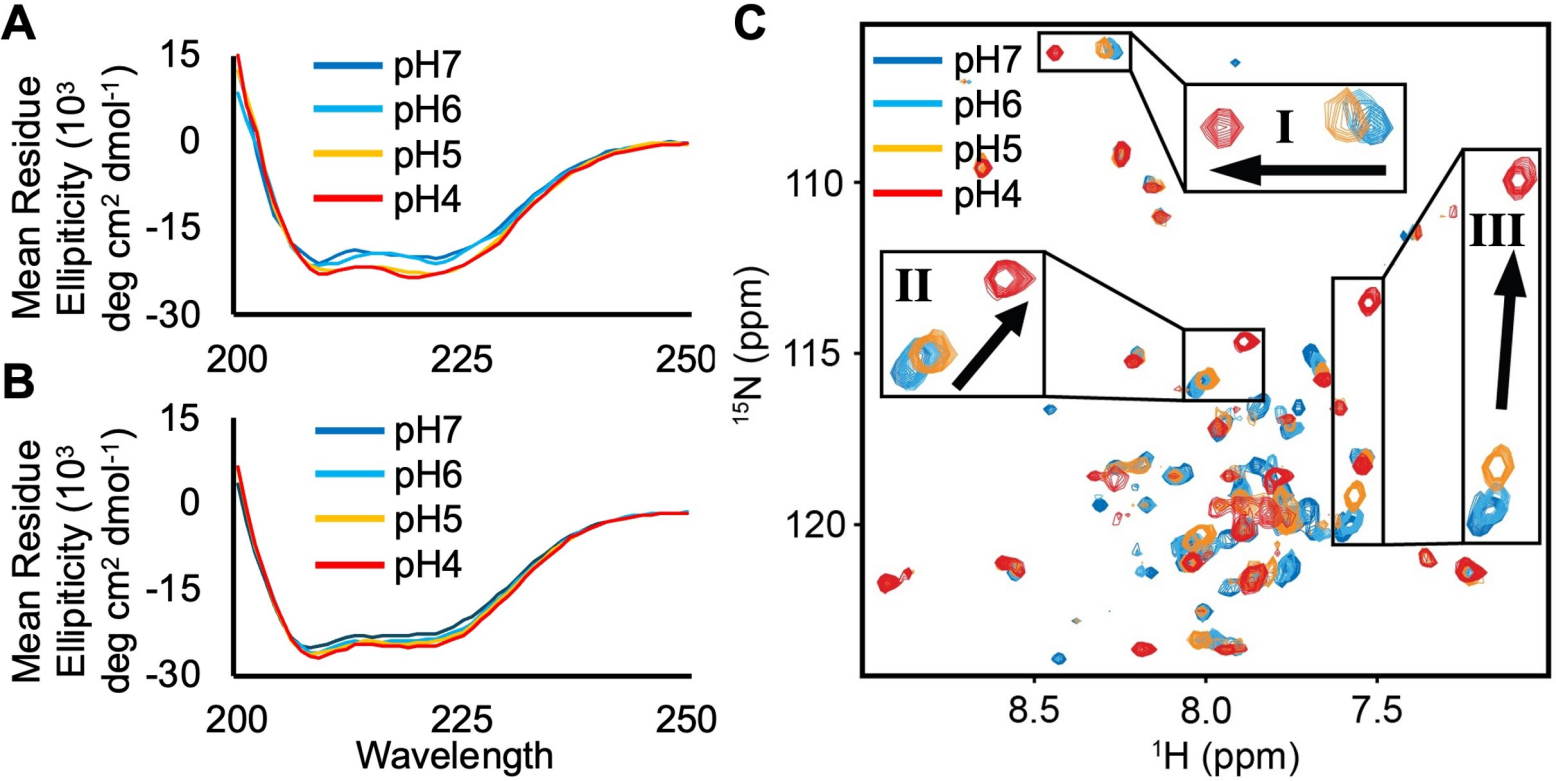
CD spectra of LASV SSP in (A) phospholipid liposomes and (B) LMPG micelles. Lipid composition was 75 : 25 POPC : POPG. (C) ^1^H−^15^N HSQC spectra of LASV SSP in LMPG micelles. SSP undergoes a structural change at low pH (5‐4) that is consistent with an increase in helical character.

LASV SSP is known to be an essential component in the pH‐dependent fusion process, however, its precise role in fusion remains elusive.[^17^, ^24^, ^32^] In this study, we investigated the structure of SSP under varying environmental pH levels to determine whether a conformational change is a component of this unique role, as protein structure dictates function. Using CD spectroscopy, we examined the secondary structure of SSP. At a neutral pH (pH 7) corresponding to a prefusion state, SSP exhibits high helical content in phospholipid liposomes, as marked by distinctive minima at 208 nm and 222 nm (Figure 3A). This helical content corresponds to 33 out of 57 α‐helix residues.^[51]^ When considering the partial cryo‐EM structure of SSP, which purports 26 α‐helix residues out of the 31 resdiues resolved for the NTH and TMH, we can deduce the CTD would contain about seven α‐helix residues, the rest of the CTD being unstructured. This agrees well with a recent computational model containing the SSP CTD, which predicts two helical turns corresponding to about 7–8 α‐helix residues while the rest was unstructured.^[52]^ To simulate the decreasing pH in the endocytic pathway, we progressively lowered the pH to mimic the acidic environment of the late endosome/lysosome (pH<5) where the LASV GPC will commence membrane fusion and, therefore, SSP will be in a postfusion state. As the pH decreased, the CD spectra showed lower minima at 208 nm and 222 nm, indicating an increase in helical content by approximately 8 % in liposomes and 6 % in LMPG micelles, corresponding to roughly four additional α‐helix residues.

To gain further insights into the structural change undergone by SSP at low pH, we employed solution NMR spectroscopy, which provides high resolution structural information and allows tracking of individual amino acids under different conditions to determine structural information.[^55^, ^56^] We used LMPG detergent micelles as a membrane mimic, which is commonly used for studying membrane proteins via NMR since liposomes are not feasible due to their slow tumbling rates.[^57^, ^58^, ^59^, ^60^, ^61^] Importantly, we confirmed that LMPG micelles generated the same structural transition in SSP at low pH as observed with liposomes via CD, validating the use of LMPG micelles for further structural investigation (Figure 3B). ^1^H−^15^N transverse relaxation‐optimized spectroscopy heteronuclear single quantum coherence (TROSY‐HSQC) NMR spectra were obtained for LASV SSP embedded in LMPG micelles at pH 7, pH 6, pH 5, and pH 4 (Figure 3C). The peaks are well dispersed across all pH levels, indicating SSP is well‐folded in LMPG micelles. However, when overlaying the spectra, we observed a majority of peaks undergo chemical shift perturbations (CSPs) at low pH (<5), indicating a change to the tertiary structure of SSP. This indicates the conformational change is not confined to just four amino acids predicted to be changing secondary structure via CD, and may involve large scale movements of one or more of the three regions of SSP. To track the structural change of SSP as the environmental pH is decreased, we focused on three peaks that had distinct chemical shift perturbations (highlighted in Figure 3C). From pH 7 to pH 6, peaks I, II, and III had CSP values of 0.02, 0.04, and 0.10, respectively, indicating little alterations in their chemical environment and suggesting SSP has a similar conformation at pH 7 and pH 6. From pH 7 to pH 5, the CSP values increase to 0.04, 0.1, and 0.37, while from pH 7 to pH 4, the peaks have robust CSP values, reaching 0.18, 0.51, and 2.57. These results indicate that SSP begins to undergo a conformational change at pH 5, which becomes fully apparent at pH 4. Consequently, we can conclude that LASV SSP experiences a pH‐dependent conformational change at low pH (pH<5), leading to a global increase in helical content.

### Locations of LASV SSP Associated with Low pH Conformational Change

To further examine the conformational change experienced by LASV SSP at low pH, we utilized tryptophan (Trp) fluorescence. Trp fluorescence has many qualities that make it ideal for this study; LASV SSP lacks Trp in its native sequence, allowing us to introduce Trp at locations of our choice. Trp is a natural amino acid, so perturbations to the SSP structure are minimized. Also, the fluorescence profile of Trp is sensitive to its surrounding environment, so structural changes will result in a robust readout.[^45^, ^62^, ^63^, ^64^, ^65^] Extensive research has shown a direct correlation between the dielectric constant of the environment surrounding Trp and Trp fluorescence emission λ_max_.[^63^, ^66^, ^67^] Specifically, polar environments result in a longer emission λ_max_, i. e. red‐shifted, while non‐polar environments result in a shorter emission λ_max_, i. e. blue‐shifted. For reference, Trp exposed to an aqueous environment, such as those on the protein surface or in an unfolded protein, reliably has an emission λ_max_ of ~350 nm.[^46^, ^65^, ^68^]

**Figure 4:**
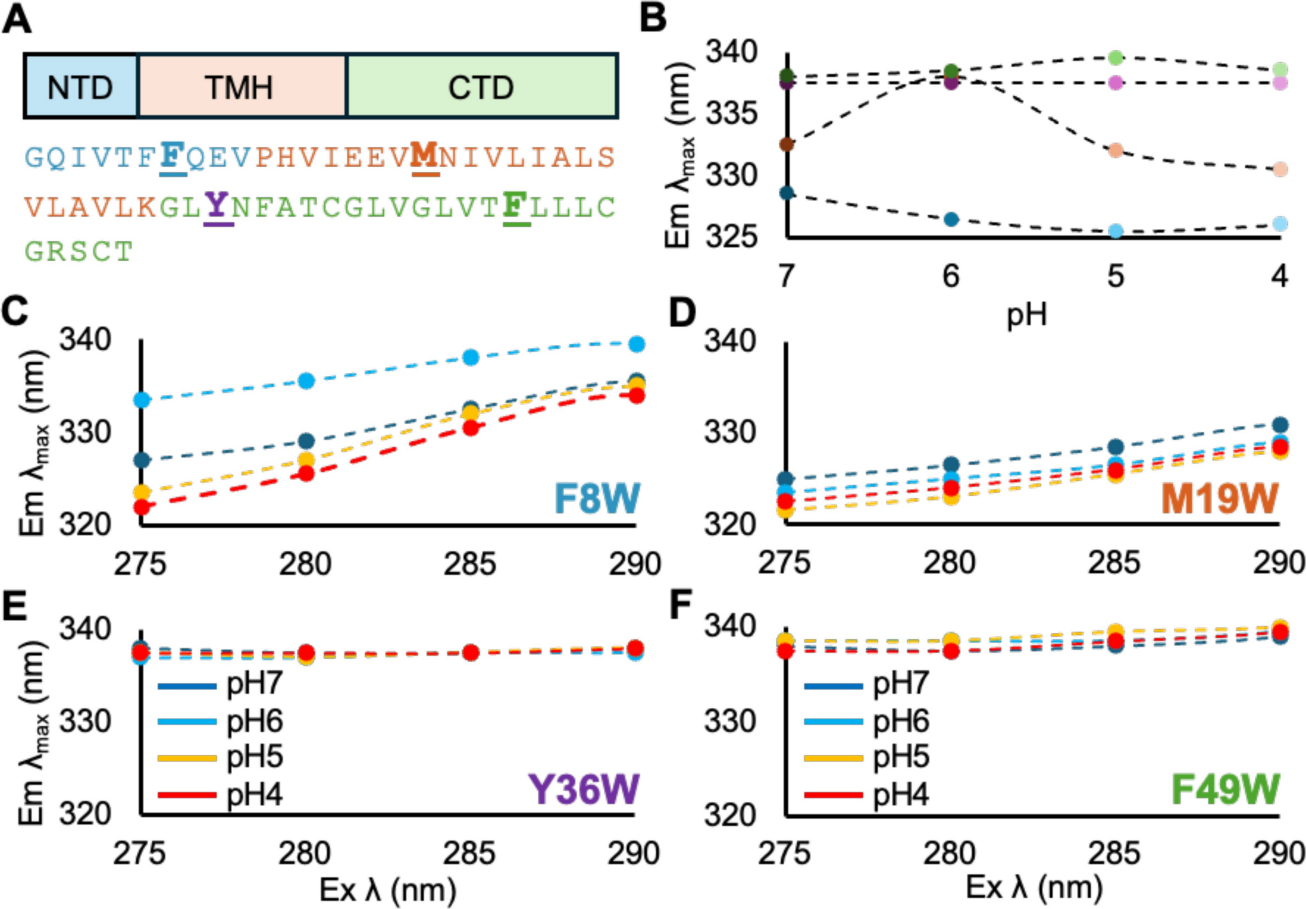
(A) Top: Distinct regions of SSP. Bottom: Locations of TRP mutant made throughout the LASV SSP sequence are shown bolded and underlined. Trp mutant color code used for B–F. (B) Emission λ_max_ for each TRP mutant embedded in liposomes plotted against pH. (C–F) red‐edge excitaion shift for each TRP mutant. The association of both NTH and TMH with the membrane increases at low pH (5–4), while the CTD is exposed to solvent at all pH levels. Lipid compositions were 75 : 25 POPC : POPG.

We selected four locations dispersed throughout the SSP sequence to make single point Trp mutants, making F8W, M19W, Y36W, and F49W mutants (Figure 4A). These locations were chosen based on two criteria. The first is that they contain either aromatic or bulky/hydrophobic amino acids to minimize the potential for structural perturbation caused by introducing Trp. Second, each location is part of a distinct structural region of SSP, namely the NTH, TMH, and CTD.[^9^, ^17^] We confirmed a lack of perturbation by the mutations via CD; the Trp mutants displayed the same amount of helicity as wild‐type SSP and experienced a similar secondary structure change at low pH (Figure S4). The emission spectrum of each Trp mutant embedded in liposomes at pH 7 were all blue‐shifted compared to Trp in an aqueous environment (~350 nm); F8W is 333 nm, M19W is 329 nm, Y36W and F49W are both 338 nm, indicating each residue resides in a more non‐polar environment (Figure 4B). Since SSP is a membrane protein, the blue‐shifted emission λ_max_ is most likely due to the Trp residues interacting with or being buried in the lipid bilayer of the liposome.[^46^, ^65^, ^69^] When viewing the different SSP regions in more detail, we observe that the NTH is an amphipathic helix, enabling it to interact with the polar head groups/aqueous solvent on one face and the hydrophobic tails on the other face (Figure S5A).^[70]^ The TMH contains mostly hydrophobic residues at its core and polar/charged residues at its N‐ and C‐termini, allowing it to interact with the hydrophobic tails and polar head groups of the lipids while it spans the bilayer (Figure S5B). Meanwhile, the CTD is posited to form a zinc‐binding complex with the cytoplasmic tail of GP2.[^33^, ^34^] This interaction would occur in the cytoplasm near the inner leaflet of the viral membrane, meaning the mostly unstructured CTD could spend some time interacting with the lipid headgroups of the bilayer.

Upon performing a pH titration to pH 4, the emission λ_max_ of F8W and M19W underwent further blue‐shifting (Figure 4B). This indicates that the environment around F8W and M19W became more non‐polar at low pH, which could be due to an increased association by SSP with the lipid bilayer.^[71]^ Therefore, we believe the regions corresponding to F8W and M19W, the NTD and TMH, respectively, are undergoing a conformational change due to low pH. As an amphipathic helix, the NTH would likely bury deeper into the membrane perpendicular to the lipids, while the bilayer spanning TMH could increase its tilt angle relative to the membrane. Meanwhile, Y36W and F49W, both a part of the CTD, remained in the same environment at low pH as indicated by the lack of λ_max_ shift. Therefore, we predict the structure of CTD does not undergo a significant structural change at low pH. This would leave the CTD exposed to the viral cytoplasm during the fusion process, likely meaning the zinc‐binding complex will remain intact while fusion is occurring.

To further understand the nature of SSP structure and the conformational change it undergoes at low pH, the red edge excitation shift (REES) of the Trp mutants was examined. REES occurs when Trp is in a motionally restricted environment, resulting in a longer emission λ_max_ (red‐shift) when excited at longer wavelengths.[^46^, ^72^, ^73^, ^74^, ^75^] At pH 7, we observed varying measures of REES for each Trp mutant; Y36W and F49W (CTD) had 0 nm and 1 nm of REES, which can be considered not significant, while F8W (NTH) and M19W (TMH) underwent 9 nm and 6 nm of REES, which is a significant amount (Figure 4C–F). Thus, Y36W and F49W must be exposed to bulk aqueous solvent due to their low REES values. However, when considering the emission λ_max_ results, Y36W and F49W likely interacts with the lipid head group region of the bilayer. Therefore, we suggest Y36W and F49W are a part of a dynamically active portion of SSP that switches between both the membrane and aqueous environment. On the other hand, F8W and M19W are in motionally restricted environments due to the high amount of REES and are likely buried within the motionally restricted environment of the lipid bilayer, which rationalizes their blue‐shifted λ_max_ values. After performing a pH titration to low pH, the amount of REES for M19W, Y36W, and F49W remained relatively unchanged, while the REES for F8W increased to 12 nm, suggesting NTH may be embedded deeper into the rigid bilayer during the fusion process. Taken together, the Trp fluorescence data further points towards the pH‐dependent conformational change of SSP being confined to the NTH and TMH. Fluorescence data is summarized in Table S1.

### Interactions between LASV GP2 and SSP Increase Fusion Efficiency

**Figure 5:**
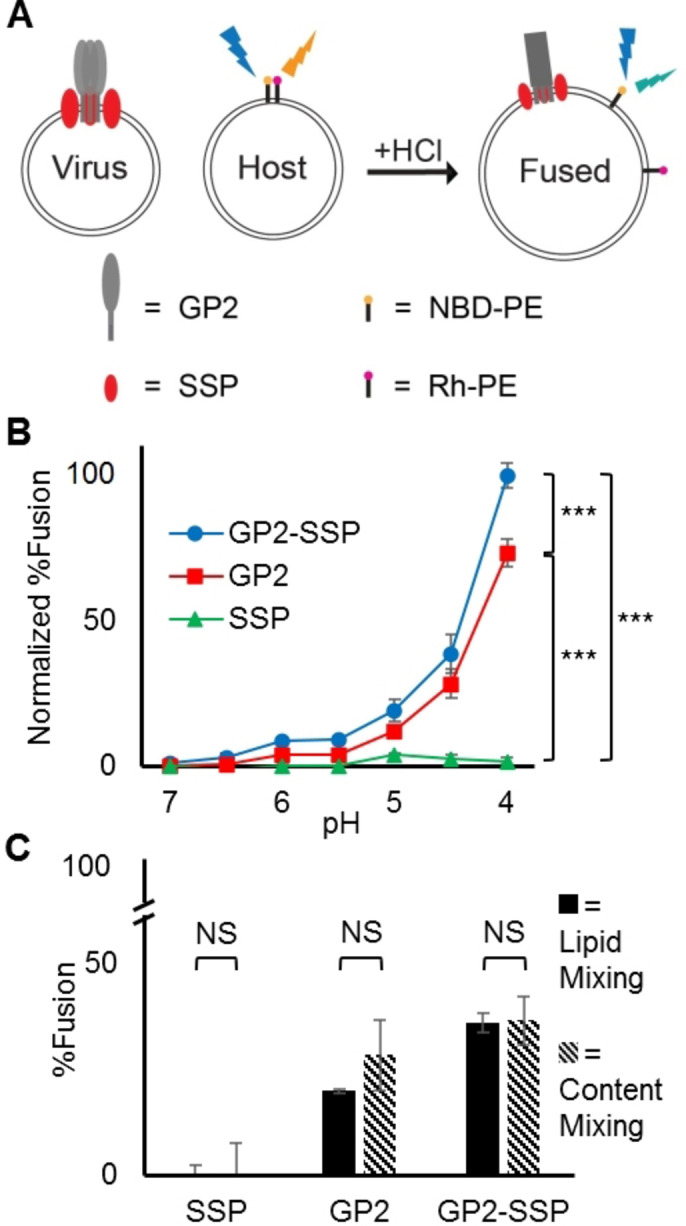
(A) Illustration of our designed lipid mixing assay. (B) Lipid mixing results for SSP, GP2, and GP2‐SSP. Lipid composition was 75 : 25 POPC : POPG. Data is normalized to GP2‐SSP fusion at pH 4, where maximum fusion is witnessed. GP2‐SSP generates more fusion than GP2 alone, while SSP alone generates minimal fusion. (C) A comparison of lipid mixing and content mixing shows no significant difference between the amount of fusion witnessed. Student's t‐Test was used to calculate P‐values; NS=not significant *=P<0.5, **=<0.01, ***=P<0.0001. n≥2.

SSP is a unique and essential component of the LASV pH‐dependent fusion mechanism that has previously been found to be crucial for efficient fusion. To explore the role of SSP in the fusion process, we have developed a modified version of a lipid mixing assay used extensively to measure the fusogenicity of viral fusion peptides to investigate the effect of SSP on LASV fusion (Figure 5A).[^36^, ^76^, ^77^, ^78^] The key advantage with this assay is it utilizes the full‐length GP2 to generate fusion, allowing for the capture of physiologically relevant fusion data in the presence of SSP. Our results show that both GP2 alone and GP2‐SSP elicit minimal fusion activity at pH levels corresponding to the host plasma membrane and early lysosome (pH 7–5.5) (Figure 5B). At these pH levels, both GP2 and GP2‐SSP generated less than 10 % relative fusion. In contrast, fusion readily occurred for both GP2 alone and GP2‐SSP at a pH environment corresponding to a late endosome/lysosome (pH<5), which agrees well with previous *in vivo* findings (Figure 5B).[^17^, ^18^, ^29^, ^79^] This demonstrates that LASV preferentially initiates fusion at this cellular compartment. In these pH environments, GP2‐SSP generates significantly more fusion than GP2, corresponding to an approximately 1.5‐fold increase in fusion at each pH level. Both GP2 alone and GP2‐SSP experience the highest level of fusion at pH 4, which is slightly lower than the pH of maximal fusion observed by others. This could be attributed to the specific pH environment of the host cell or other host factors, particularly the presence of the endosomal receptor LAMP1, which may allow the conformational change of GPC, and importantly GP2, to occur at a slightly elevated pH. Research has shown that LAMP1 promotes LASV fusion in less acidic environments, which may explain the lower pH of maximum fusion observed in our assay.[^10^, ^11^, ^12^, ^80^]

Interestingly, SSP alone produced insignificant levels of fusion across all tested pH levels (Figure 5B). This suggests that the increase in fusion observed by GP2 in the presence of SSP is likely due to interactions formed between the two, rather than SSP independently driving the fusion process. These findings confirm that SSP increases the fusion efficiency of GP2 at lower pH levels, potentially by facilitating GP2 into a fusion‐ready state through interactions formed between the two. Previous studies have demonstrated that interactions between SSP and GP2 are crucial for LASV fusion, with mutations to residues predicted to be involved in these interactions resulting in reduced fusion efficiency.[^17^, ^26^, ^32^] Furthermore, since SSP undergoes a structural change in an acidic environment, it is plausible that this structural change is directly correlated with the ability of SSP to increase fusion.

We ran a content mixing assay to better understand the fusion data obtained from our assay. The advantage of a content mixing assay is its signal is linked to an increase in volume of the liposome cavity and, therefore, is indicative of a full fusion event in our assay.^[81]^ Within each sample tested (e. g. SSP, GP2, GP2‐SSP) we observed no significant difference between the amount of fusion generated in the content mixing and lipid mixing assays (Figure 5C). Therefore, the signal in our lipid mixing assay is due to full fusion and rules out the possibility of other events like liposome bursting, docking, or hemifusion, which would all lead to positive signals in lipid mixing but are accounted for in content mixing.[^81^, ^82^, ^83^]

### Model of LASV SSP Conformational Change During Fusion

**Figure 6:**
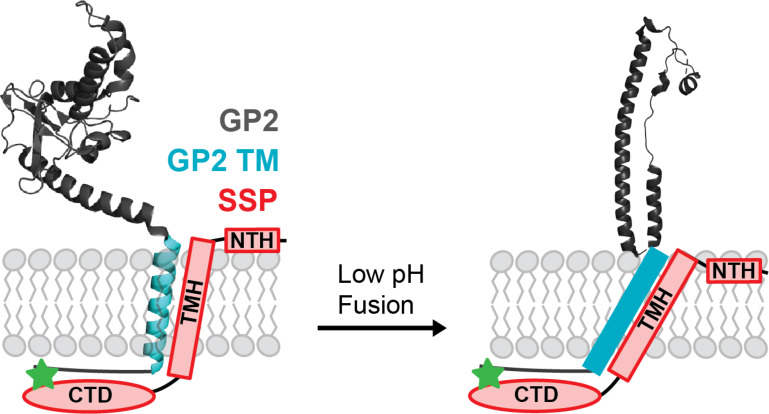
Predicted structural changes undergone by LASV SSP during the fusion process. GP2 structures taken from the PDBs 7PUYand 5OMI. Green star represents zinc‐binding complex between CTD of SSP and cytoplasmic tail of GP2.

We have developed a model to illustrate how the structure of SSP changes within the membrane in response to low pH (Figure 6). When the pH drops below 5, the N‐terminal helix (NTH) appears to embed deeper into the membrane bilayer, while concurrently, the transmembrane helix (TMH) likely increases its tilt angle. Meanwhile, the C‐terminal domain (CTD) of SSP remains unchanged during the fusion process as it forms a crucial zinc‐binding complex with the cytoplasmic tail of GP2.[^33^, ^34^] Disruption of this inter‐subunit zinc‐binding complex has been shown to negatively impact GPC surface expression and mature virion formation, indicating that the CTD plays a crucial role earlier in the LASV lifecycle, ensuring that the virion assembles correctly before it can fuse with host cells.[^24^, ^84^]

The close relationship between SSP and GP2 is crucial for LASV virions to infect host cells. The interface between TMH of SSP and TM of GP2 has been identified as the location where small molecule fusion inhibitors of LASV bind, which underscores how critical their interactions are for fusion.[^28^, ^29^, ^30^, ^31^, ^52^] Inhibitors that target this interface could disrupt TM‐TMH interactions leading to abnormalities including premature conformation changes or the stabilization of a non‐functional state, both scenerios would effectively block fusion. These are consistent with the mechanism of small molecule fusion inhibitors used against related viruses; inhibitors against Ebola virus cause destabilization of the prefusion state causing premature conformational changes, while inhibitors against HIV and influenza mainly act to stabilize the prefusion state of the fusion protein thereby preventing the necessary conformational rearrangement to accomplish fusion.[^85^, ^86^, ^87^, ^88^, ^89^, ^90^]

In LASV and other related viruses, like HIV or Ebola virus, the membrane‐proximal external region (MPER) and TM of GP2 have been identified as contributors toward fusion efficiency.[^36^, ^91^, ^92^, ^93^, ^94^, ^95^, ^96^] In LASV, these regions are thought to interact closely with the NTH and TMH of SSP, the regions we have identified undergoing a conformational change during fusion.[^9^, ^30^, ^32^] Additionally, the TM of LASV undergoes a helical extension at low pH that was found to be crucial towards LASV fusion efficiency. This extension likely increases the tilt angle relative to the membrane, preventing hydrophobic mismatch and ensuring proper membrane fusion.[^36^, ^47^] Similar changes in tilt angle have been observed in the fusion activity of other related viruses.[^96^, ^97^] Thus, it is possible the TMH of SSP undergoes a helical extension, leading to a similar increase in tilt, which could help maintain its interaction with the TM of GP2 during the fusion process.

Our research indicates that the structural changes in the NTH and TMH of SSP are crucial for its interactions with GP2, directly driving the fusion process in LASV. Investigation of these key residues will help us further understand how LASV enters host cells and highlight potential targets for therapeutic intervention. By focusing on disrupting these critical interactions or stabilizing specific protein conformations, we may be able to prevent LASV virions from fusing with host cells, effectively blocking infection.

## Conclusions

SSP is a unique component of the arenavirus GPC that plays a critical, yet mysterious role in LASV membrane fusion. Our findings reveal that SSP undergoes a conformational change in the acidic environment of a late endosome/lysosome, which is associated with enhanced GP2‐mediated membrane fusion. Specifically, we identified that the N‐terminal helix (NTH) and transmembrane helix (TMH) regions of SSP undergo a conformational change. Future research should investigate this conformational change in more detail, particularly focusing on how the conformational change of SSP influences GP2‐SSP interactions that are critical for LASV fusion. The *in vitro* methods developed in this study provide a foundation for further exploration of GP2‐SSP interactions, key SSP residues, and screening small molecule inhibitors of LASV fusion. This investigation will further unlock insight into the role and evolutionary advantage of SSP in the LASV fusion mechanism.

## Funding

This work was supported by startup funds from the University of Maryland – College Park.

## Author Contributions

S. C. and J. L. designed the experiments. S. C. performed and analyzed DLS, CD, NMR, Trp fluorescence, and lipid mixing experiments, and L.J. performed and analyzed Cryo‐EM. S. C., Y. L., and J. L. prepared the manuscript.

## Conflict of Interests

The authors declare no conflict of interest.

1

## Supporting information

As a service to our authors and readers, this journal provides supporting information supplied by the authors. Such materials are peer reviewed and may be re‐organized for online delivery, but are not copy‐edited or typeset. Technical support issues arising from supporting information (other than missing files) should be addressed to the authors.

Supporting Information

## Data Availability

The data that support the findings of this study are available from the corresponding author upon reasonable request.

## References

[1] O. Ogbu , E. Ajuluchukwu , C. J. Uneke , J. Vector Borne Dis. 2007, 44, 1–11. 17378212

[2] C. Aloke , N. A. Obasi , P. M. Aja , C. U. Emelike , C. O. Egwu , O. Jeje , C. O. Edeogu , O. O. Onisuru , O. U. Orji , I. Achilonu , Viruses 2023, 15, 146. 36680186 10.3390/v15010146 PMC9864412

[3] “Pathogens prioritization: a scientific framework for epidemic and pandemic research preparedness,” can be found under https://www.who.int/publications/m/item/pathogens-prioritization-a-scientific-framework-for-epidemic-and-pandemic-research-preparedness, n.d.

[4] D. J. Burri , J. R. da Palma , S. Kunz , A. Pasquato , Viruses 2012, 4, 2162–2181. 23202458 10.3390/v4102162 PMC3497046

[5] K. A. Willard , J. T. Alston , M. Acciani , M. A. Brindley , Pathogenesis 2019, 8, 1. 10.3390/pathogens8010001 PMC6471855 30587764

[6] H. N. Pennington , J. Lee , Biosci. Rep. 2022, 42, BSR20211930. 35088070 10.1042/BSR20211930 PMC8844875

[7] M. Acciani , J. T. Alston , G. Zhao , H. Reynolds , A. M. Ali , B. Xu , M. A. Brindley , J. Virol. 2017, 91, e00574–17. 28679759 10.1128/JVI.00574-17 PMC5571257

[8] S. Kunz , J. M. Rojek , M. Perez , C. F. Spiropoulou , M. B. A. Oldstone , J. Virol. 2005, 79, 5979–5987. 15857984 10.1128/JVI.79.10.5979-5987.2005 PMC1091707

[9] M. Katz , J. Weinstein , M. Eilon-Ashkenazy , K. Gehring , H. Cohen-Dvashi , N. Elad , S. J. Fleishman , R. Diskin , Nature 2022, 603, 174–179. 35173332 10.1038/s41586-022-04429-2

[10] H. Cohen-Dvashi , N. Cohen , H. Israeli , R. Diskin , J. Virol. 2015, 89, 7584–7592. 25972533 10.1128/JVI.00651-15 PMC4505663

[11] H. Cohen-Dvashi , H. Israeli , O. Shani , A. Katz , R. Diskin , J. Virol. 2016, 90, 10329–10338. 27605678 10.1128/JVI.01624-16 PMC5105667

[12] S. Li , Z. Sun , R. Pryce , M. L. Parsy , S. K. Fehling , K. Schlie , C. A. Siebert , W. Garten , T. A. Bowden , T. Strecker , J. T. Huiskonen , PLoS Pathog. 2016, 12, e1005418. 26849049 10.1371/journal.ppat.1005418 PMC4743923

[13] S. A. Gallo , A. Puri , R. Blumenthal , Biochemistry 2001, 40, 12231–12236. 11591141 10.1021/bi0155596

[14] D. J. Benton , S. J. Gamblin , P. B. Rosenthal , J. J. Skehel , Nature 2020, 583, 150–153. 32461688 10.1038/s41586-020-2333-6 PMC7116728

[15] J. Lee , A. J. B. Kreutzberger , L. Odongo , E. A. Nelson , D. A. Nyenhuis , V. Kiessling , B. Liang , D. S. Cafiso , J. M. White , L. K. Tamm , Nat. Struct. Mol. Biol. 2021, 28, 181–189. 33462517 10.1038/s41594-020-00548-4 PMC7992113

[16] S. C. Harrison , Nat. Struct. Mol. Biol. 2008, 15, 690–698. 18596815 10.1038/nsmb.1456 PMC2517140

[17] E. L. Messina , J. York , J. H. Nunberg , J. Virol. 2012, 86, 6138–6145. 22438561 10.1128/JVI.07241-11 PMC3372177

[18] J. York , J. H. Nunberg , J. Virol. 2006, 80, 7775–7780. 16840359 10.1128/JVI.00642-06 PMC1563716

[19] L. H. Bederka , C. J. Bonhomme , E. L. Ling , M. J. Buchmeier , mBio 2014, 5, e02063–14. 25352624 10.1128/mBio.02063-14 PMC4217180

[20] S. S. Agnihothram , J. York , J. H. Nunberg , J. Virol. 2006, 80, 5189–5198. 16698999 10.1128/JVI.00208-06 PMC1472124

[21] R. Eichler , O. Lenz , T. Strecker , M. Eickmann , H. Klenk , W. Garten , EMBO Rep. 2003, 4, 1084–1088. 14555961 10.1038/sj.embor.7400002 PMC1326372

[22] H. Owji , N. Nezafat , M. Negahdaripour , A. Hajiebrahimi , Y. Ghasemi , Eur. J. Cell Biol. 2018, 97, 422–441. 29958716 10.1016/j.ejcb.2018.06.003

[23] J. York , V. Romanowski , M. Lu , J. H. Nunberg , J. Virol. 2004, 78, 10783–10792. 15367645 10.1128/JVI.78.19.10783-10792.2004 PMC516395

[24] A. A. Saunders , J. P. C. Ting , J. Meisner , B. W. Neuman , M. Perez , J. C. de la Torre , M. J. Buchmeier , J. Virol. 2007, 81, 5649–5657. 17376927 10.1128/JVI.02759-06 PMC1900251

[25] J. York , J. H. Nunberg , Virology 2007, 359, 72–81. 17045626 10.1016/j.virol.2006.08.048

[26] J. York , J. H. Nunberg , J. Virol. 2009, 83, 4121–4126. 19224989 10.1128/JVI.02410-08 PMC2668491

[27] J. York , J. H. Nunberg , J. Virol. 2017, 92, e01682–17.29070682

[28] A. M. Lee , J. M. Rojek , C. F. Spiropoulou , A. T. Gundersen , W. Jin , A. Shaginian , J. York , J. H. Nunberg , D. L. Boger , M. B. A. Oldstone , S. Kunz , J. Biol. Chem. 2008, 283, 18734–18742. 18474596 10.1074/jbc.M802089200 PMC2441566

[29] J. York , D. Dai , S. M. Amberg , J. H. Nunberg , J. Virol. 2008, 82, 10932–10939. 18768973 10.1128/JVI.01140-08 PMC2573205

[30] C. J. Thomas , H. E. Casquilho-Gray , Y. Joanne , D. L. DeCamp , D. Dongcheng , E. B. Petrilli , D. L. Boger , R. A. Slayden , S. M. Amberg , S. R. Sprang , J. H. Nunberg , J. Biol. Chem. 2011, 286, 6192–6200. 21159779 10.1074/jbc.M110.196428 PMC3057843

[31] R. A. Larson , D. Dai , V. T. Hosack , Y. Tan , T. C. Bolken , D. E. Hruby , S. M. Amberg , J. Virol. 2008, 82, 10768–10775. 18715909 10.1128/JVI.00941-08 PMC2573164

[32] S. Shankar , L. R. Whitby , H. E. Casquilho-Gray , J. York , D. L. Boger , J. H. Nunberg , J. Virol. 2016, 90, 6799–6807. 27194767 10.1128/JVI.00597-16 PMC4944282

[33] K. Briknarová , C. J. Thomas , J. York , J. H. Nunberg , J. Biol. Chem. 2011, 286, 1528–1536. 21068387 10.1074/jbc.M110.166025 PMC3020761

[34] J. York , J. H. Nunberg , J. Virol. 2007, 81, 13385–13391. 17928348 10.1128/JVI.01785-07 PMC2168868

[35] C. Diefenderfer , J. Lee , S. Mlyanarski , Y. Guo , K. J. Glover , Anal. Biochem. 2009, 384, 274–278. 18929529 10.1016/j.ab.2008.09.038

[36] P. M. Keating , N. P. Schifano , X. Wei , M. Y. Kong , J. Lee , Biochim. Biophys. Acta BBA – Biomembr. 2024, 1866, 184233. 10.1016/j.bbamem.2023.184233 37734457

[37] A. J. Miles , B. A. Wallace , Protein Sci. Publ. Protein Soc. 2018, 27, 1717–1722. 10.1002/pro.3474 PMC6194270 30168221

[38] D. Birtles , J. Lee , Biochemistry 2021, 60, 2978–2986. 34570469 10.1021/acs.biochem.1c00543

[39] A. J. Miles , S. G. Ramalli , B. A. Wallace , Protein Sci. 2022, 31, 37–46. 34216059 10.1002/pro.4153 PMC8740839

[40] L. A. Compton , W. C. Johnson , Anal. Biochem. 1986, 155, 155–167. 3717552 10.1016/0003-2697(86)90241-1

[41] J. Marley , M. Lu , C. Bracken , J. Biomol. NMR 2001, 20, 71–75. 11430757 10.1023/a:1011254402785

[42] F. Delaglio, S. Grzesiek, GeertenW. Vuister, G. Zhu, J. Pfeifer, A. Bax, *J. Biomol. NMR* **1995**, *6*, 277-293 DOI: 10.1007/BF00197809. 8520220

[43] W. Lee , M. Tonelli , J. L. Markley , Bioinformatics 2015, 31, 1325–1327. 25505092 10.1093/bioinformatics/btu830 PMC4393527

[44] M. W. Maciejewski , A. D. Schuyler , M. R. Gryk , I. I. Moraru , P. R. Romero , E. L. Ulrich , H. R. Eghbalnia , M. Livny , F. Delaglio , J. C. Hoch , Biophys. J. 2017, 112, 1529–1534. 28445744 10.1016/j.bpj.2017.03.011 PMC5406371

[45] R. K. Kamlekar , Y. Gao , R. Kenoth , J. G. Molotkovsky , F. G. Prendergast , L. Malinina , D. J. Patel , W. S. Wessels , S. Y. Venyaminov , R. E. Brown , Biophys. J. 2010, 99, 2626–2635. 20959104 10.1016/j.bpj.2010.08.038 PMC2955354

[46] S. Alim , S. Das , M. J. Swamy , J. Photochem. Photobiol. C 2023, 440, 114643.

[47] P. M. Keating , H. N. Pennington , S. D. Collins , J. Lee , Biochem. Biophys. Rep. 2022, 33, 101409. 36583076 10.1016/j.bbrep.2022.101409 PMC9792740

[48] L. Tonggu , L. Wang , Ultramicroscopy 2020, 208, 112849. 31622807 10.1016/j.ultramic.2019.112849 PMC7058178

[49] X. Yao , X. Fan , N. Yan , Proc. Natl. Acad. Sci. USA 2020, 117, 18497–18503. 32680969 10.1073/pnas.2009385117 PMC7414195

[50] C. H. Crouch , M. H. Bost , T. H. Kim , B. M. Green , D. S. Arbuckle , C. H. Grossman , K. P. Howard , Membranes 2018, 8, 103. 30413063 10.3390/membranes8040103 PMC6315538

[51] A. J. Miles , B. A. Wallace , Chem. Soc. Rev. 2016, 45, 4859–4872. 27347568 10.1039/c5cs00084j

[52] Z. Zhang , T. Takenaga , S. K. Fehling , M. Igarashi , T. Hirokawa , Y. Muramoto , K. Yamauchi , C. Onishi , M. Nakano , S. Urata , A. Groseth , T. Strecker , T. Noda , J. Virol. 2024, 98, e00714–24. 38809021 10.1128/jvi.00714-24 PMC11265444

[53] M. Johnsson , K. Edwards , Biophys. J. 2003, 85, 3839–3847. 14645073 10.1016/S0006-3495(03)74798-5 PMC1303685

[54] P. Xie , H. Zhang , Y. Qin , H. Xiong , C. Shi , Z. Zhou , Biomol. Eng. 2023, 13, 1772. 10.3390/biom13121772 PMC10741411 38136643

[55] S. J. Opella , F. M. Marassi , Arch. Biochem. Biophys. 2017, 628, 92–101. 28529197 10.1016/j.abb.2017.05.011 PMC5657258

[56] R. Puthenveetil , O. Vinogradova , J. Biol. Chem. 2019, 294, 15914–15931. 31551353 10.1074/jbc.REV119.009178 PMC6827292

[57] W. Surya , Y. Li , J. Torres , Biochim. Biophys. Acta Biomembr. 2018, 1860, 1309–1317. 29474890 10.1016/j.bbamem.2018.02.017 PMC7094280

[58] C. Tian , C. G. Vanoye , C. Kang , R. C. Welch , H. J. Kim , A. L. George , C. R. Sanders , Biochemistry 2007, 46, 11459–11472. 17892302 10.1021/bi700705j PMC2565491

[59] A. J. Beel , C. K. Mobley , H. J. Kim , F. Tian , A. Hadziselimovic , B. Jap , J. H. Prestegard , C. R. Sanders , Biochemistry 2008, 47, 9428–9446. 18702528 10.1021/bi800993c PMC2572687

[60] T. Zhuang , B. K. Jap , C. R. Sanders , J. Am. Chem. Soc. 2011, 133, 20571–20580. 22084929 10.1021/ja208972h PMC3241871

[61] I. Maslennikov , C. Klammt , E. Hwang , G. Kefala , M. Okamura , L. Esquivies , K. Mörs , C. Glaubitz , W. Kwiatkowski , Y. H. Jeon , S. Choe , Proc. Nat. Acad. Sci. 2010, 107, 10902–10907. 20498088 10.1073/pnas.1001656107 PMC2890740

[62] A. B. T. Ghisaidoobe , S. J. Chung , Int. J. Mol. Sci. 2014, 15, 22518–22538. 25490136 10.3390/ijms151222518 PMC4284722

[63] R. Kenoth , D. K. Simanshu , R. K. Kamlekar , H. M. Pike , J. G. Molotkovsky , L. M. Benson , H. R. Bergen , F. G. Prendergast , L. Malinina , S. Y. Venyaminov , D. J. Patel , R. E. Brown , J. Biol. Chem. 2010, 285, 13066–13078. 20164530 10.1074/jbc.M109.093203 PMC2857133

[64] K. M. Sanchez , J. E. Gable , D. E. Schlamadinger , J. E. Kim , Biochemistry 2008, 47, 12844–12852. 18991402 10.1021/bi800860k PMC2724591

[65] C. P. Moon , K. G. Fleming , Methods Enzymol. 2011, 492, 189–211. 21333792 10.1016/B978-0-12-381268-1.00018-5 PMC3799943

[66] J. T. Vivian , P. R. Callis , Biophys. J. 2001, 80, 2093–2109. 11325713 10.1016/S0006-3495(01)76183-8 PMC1301402

[67] M. Ghose , S. Mandal , D. Roy , R. K. Mandal , G. Basu , FEBS Lett. 2001, 509, 337–340. 11741613 10.1016/s0014-5793(01)03202-1

[68] X. Shen , J. R. Knutson , J. Phys. Chem. B 2001, 105, 6260–6265.

[69] G. A. Caputo , E. London , Biochemistry 2003, 42, 3265–3274. 12641458 10.1021/bi026696l

[70] A. R. Mól , M. S. Castro , W. Fontes , BioRxiv 2018, 416347.

[71] G. Slaybaugh , D. Weerakkody , D. M. Engelman , O. A. Andreev , Y. K. Reshetnyak , Proc. Nat. Acad. Sci. 2020, 117, 12095–12100. 32409607 10.1073/pnas.1917857117 PMC7275707

[72] A. P. Demchenko, *Eur. Biophys. J*. **1988**, *16*, DOI: 10.1007/BF00255522. 3208709

[73] A. K. Warrender , J. Pan , C. Pudney , V. L. Arcus , W. Kelton , J. R. Soc. Interface 2023, 20, 20230337. 37935360 10.1098/rsif.2023.0337 PMC10645072

[74] A. Chattopadhyay , S. Haldar , Acc. Chem. Res. 2014, 47, 12–19. 23981188 10.1021/ar400006z

[75] G. Maglia , A. Jonckheer , M. De Maeyer , J.-M. Frère , Y. Engelborghs , Protein Sci. Publ. Protein Soc. 2008, 17, 352–361. 10.1110/ps.073147608 PMC2222716 18096643

[76] D. Birtles , A. E. Oh , J. Lee , Protein Sci. 2022, 31, DOI: 10.1002/pro.4390.

[77] A. L. Lai , A. E. Moorthy , Y. Li , L. K. Tamm , J. Mol. Biol. 2012, 418, DOI: 10.1016/j.jmb.2012.02.010. PMC3654243 22343048

[78] S. M. Gregory , E. Harada , B. Y. Liang , S. E. Delos , J. M. White , L. K. Tamm , Proc. Natl. Acad. Sci. USA 2011, 108, 11211–11216. 21690393 10.1073/pnas.1104760108 PMC3131375

[79] H. N. Pennington , D. Birtles , Z. W. Shi , J. Lee , ACS Omega 2024, 9, 4920–4930. 38313535 10.1021/acsomega.3c08632 PMC10831964

[80] C. E. Hulseberg , L. Fénéant , K. M. Szymańska , J. M. White , mBio 2018, 9, e01818–17. 29295909 10.1128/mBio.01818-17 PMC5750398

[81] X. Liu , A. B. Seven , J. Xu , V. Esser , L. Su , C. Ma , J. Rizo , Nat. Protoc. 2017, 12, 2014–2028. 28858288 10.1038/nprot.2017.068 PMC6163043

[82] A. Cypionka , A. Stein , J. M. Hernandez , H. Hippchen , R. Jahn , P. J. Walla , Proc. Nat. Acad. Sci. 2009, 106, 18575–18580. 19843696 10.1073/pnas.0906677106 PMC2764736

[83] C. François-Martin , F. Pincet , Sci. Rep. 2017, 7, 43860. 28266607 10.1038/srep43860 PMC5339690

[84] J. Shao , X. Liu , H. Ly , Y. Liang , J. Virol. 2016, 90, 10390–10397. 27630230 10.1128/JVI.01154-16 PMC5105672

[85] H.-Y. Liu , P. L. Yang , Annu. Rev. Virol. 2021, 8, 459–489. 34197186 10.1146/annurev-virology-022221-063725 PMC8543812

[86] T. Xiao , G. Frey , Q. Fu , C. L. Lavine , D. A. Scott , M. S. Seaman , J. J. Chou , B. Chen , Nat. Chem. Biol. 2020, 16, 529–537. 32152540 10.1038/s41589-020-0496-y PMC7723321

[87] Q. Fu , M. M. Shaik , Y. Cai , F. Ghantous , A. Piai , H. Peng , S. Rits-Volloch , Z. Liu , S. C. Harrison , M. S. Seaman , B. Chen , J. J. Chou , Proc. Natl. Acad. Sci. USA 2018, 115, E8892–E8899. 30185554 10.1073/pnas.1807259115 PMC6156635

[88] R. J. Russell , P. S. Kerry , D. J. Stevens , D. A. Steinhauer , S. R. Martin , S. J. Gamblin , J. J. Skehel , Proc. Natl. Acad. Sci. USA 2008, 105, 17736–17741. 19004788 10.1073/pnas.0807142105 PMC2584702

[89] I. A. Leneva , R. J. Russell , Y. S. Boriskin , A. J. Hay , Antiviral Res. 2009, 81, 132–140. 19028526 10.1016/j.antiviral.2008.10.009

[90] Y. Zhao , J. Ren , K. Harlos , D. M. Jones , A. Zeltina , T. A. Bowden , S. Padilla-Parra , E. E. Fry , D. I. Stuart , Nature 2016, 535, 169–172. 27362232 10.1038/nature18615 PMC4947387

[91] J. Cao , G. Zhang , M. Zhou , Y. Liu , G. Xiao , W. Wang , Virol. Sin. 2021, 36, 273–280. 32897505 10.1007/s12250-020-00286-3 PMC8087742

[92] C. T. Barrett , R. E. Dutch , Viruses 2020, 12, 693.32604992

[93] A. S. Dimitrov , S. S. Rawat , S. Jiang , R. Blumenthal , Biochemistry 2003, 42, 14150–14158. 14640682 10.1021/bi035154g

[94] Z. Gong , S. A. Kessans , L. Song , K. Dörner , H. Lee , L. R. Meador , J. LaBaer , B. G. Hogue , T. S. Mor , P. Fromme , Protein Sci. 2014, 23, 1607–1618. 25155369 10.1002/pro.2540 PMC4241111

[95] J. Lee , D. A. Nyenhuis , E. A. Nelson , D. S. Cafiso , J. M. White , L. K. Tamm , Proc. Nat. Acad. Sci. 2017, 114, E7987–E7996. 28874543 10.1073/pnas.1708052114 PMC5617291

[96] J. M. White , A. E. Ward , L. Odongo , L. K. Tamm , Annu. Rev. Virol. 2023, 10, 139–161. 37774128 10.1146/annurev-virology-111821-093413 PMC10866366

[97] D. J. Benton , A. Nans , L. J. Calder , J. Turner , U. Neu , Y. P. Lin , E. Ketelaars , N. L. Kallewaard , D. Corti , A. Lanzavecchia , S. J. Gamblin , P. B. Rosenthal , J. J. Skehel , Proc. Natl. Acad. Sci. USA 2018, 115, 10112–10117. 30224494 10.1073/pnas.1810927115 PMC6176637

[98] B. Apellániz , E. Rujas , S. Serrano , K. Morante , K. Tsumoto , J. M. M. Caaveiro , M. Á. Jiménez , J. L. Nieva , J. Biol. Chem. 2015, 290, 12999–13015. 25787074 10.1074/jbc.M115.644351 PMC4505554

