# Supplementary material for: The Lassa Virus Stable Signal Peptide Undergoes a Conformational Change to Aid Viral Fusion
Source: Chemistry. 2025 Mar 7;31(18):e202403608. doi: 10.1002/chem.202403608 (PMC11937871; doi:10.1002/chem.202403608)
Supplement: Supplementary file 1 — Supporting Information [file CHEM-31-e202403608-s001.pdf]

Chemistry–A European Journal

Supporting Information

The Lassa Virus Stable Signal Peptide Undergoes a Conformational Change to Aid Viral Fusion

Shane D. Collins, Liqun Jiang, Yanxin Liu, and Jinwoo Lee*

The Lassa Virus Stable Signal Peptide Undergoes a Conformational Change to Aid Viral Fusion

Shane D. Collins[1], Liqun Jiang[1,2], Yanxin Liu[1,2], and Jinwoo Lee[1,*]

1 Department of Chemistry and Biochemistry, College of Computer, Mathematics, and Natural Science, University of Maryland College Park, College Park, MD, 20740, USA
2 Institute for Bioscience and Biotechnology Research, University of Maryland, Rockville, MD, 20850, USA.

* Correspondence: Jinwoo Lee (jinwoo@umd.edu)

```
                   10        20        30        40        50
LASV      MGQIVTFFQEVPHVIEEVMNIVLIALSVLAVLKGLYNFATCGLVGLVTFLLLCGRSCT
LCMV      MGQIVTMFEALPHIIDEVINIVIIVLIVITGIKAVYNFATCGIFALISFLLLAGRSCG
LUJOV     MGQIVAVFQAIPEILNEAINIVIIVIIMFTLIKGVFNLYKSGLFQLVIFLLLCGKRCD
IPPYV     MGQIITFFQEVPHIIEEVMNIVLITLSLLAILKGVYNVMTCGLIGLISFLLLCGKSCS
MOPV      MGQIVTFFQEVPHILEEVMNIVLMTLSILAILKGIYNVMTCGIIGLITFLFLCGRSCS
MOBV      MGQIVTFFQEVPHIIEEVMNIVLITLSLLAILKGIYNVMTCGLIGLLTFLFLCGKSCS
DNDV      MGQLITMFEALPHIIDEVINIVIIVLVIITSIKAVYNFATCGIIALISFCLLAGRSCG

JUNV      MGQFISFMQEIPTFLQEALNIALVAVSLIAIIKGVVNLYKSGLFQFFVFLALAGRSCT
MACV      MGQLISFFQEIPVFLQEALNIALVAVSLIAVIKGIINLYKSGLFQFIFFLLLAGRSCS
SABV      MGQLFSFFEEVPNIIHEAINIALIAVSLIAALKGMINLWKSGLFQLIFFLTLAGRSCS
CHAVB     MGQLVSFFQEIPNIIQEAINIALIAVSLIAILKGLVNLWKSGLFQLLVFLILAGRSCS
GTOV      MGQLISFFQDIPIFFEEALNVALAVVTLLAIIKGIVNVWKSGILQLFVFLVLAGRSCS
WWAV      MGQLISFFGEIPSIIHEALNIALIAVSIISILKGVINIWGSGLLQFIVFLLLAGRSCS
PICV      MGQIVTLIQSIPEVLQEVFNVALIIVSVLCIVKGFVNLMRCGLFQLVTFLILSGRSCD
          ***:.:.:  :* .:.*.:*:.:  : ::  :*.. *.  .*:. :. *  *.*: *
```

Figure S1. Sequence alignment of Old-World (top) and New-World (bottom) arenaviruses. LCMV, Lymphocytic choriomeningitis virus; LUJOV, Lujo virus; IPPYV, Ippy virus; MOPV, Mopeia virus; MOBV, Mobala virus; DNDV, Dandenong virus; JUNV, Junin virus; MACV, Machupo virus; SABV, Sabia virus; CHAVB, Chapare virus; GTOV, Guanarito virus; WWAV, Whitewater Arroyo virus; PICV, Pichinde virus.

A

HHHHHHHHKAIFVLKGSLDRDLDSRIELELRTDHKELSEHLLLVDLARNDLARIATPGSRYVAD
LTKVDRYSYVLHLVSRVVGELRHDLDALHAYRAALNLGTLSGAPKVRAGGLVPRGQIVTFFQEVP
HVIEEVMNIVLIALSVLAVLKGLYNFATSGLVGLVTFLLLAGRSCT

B

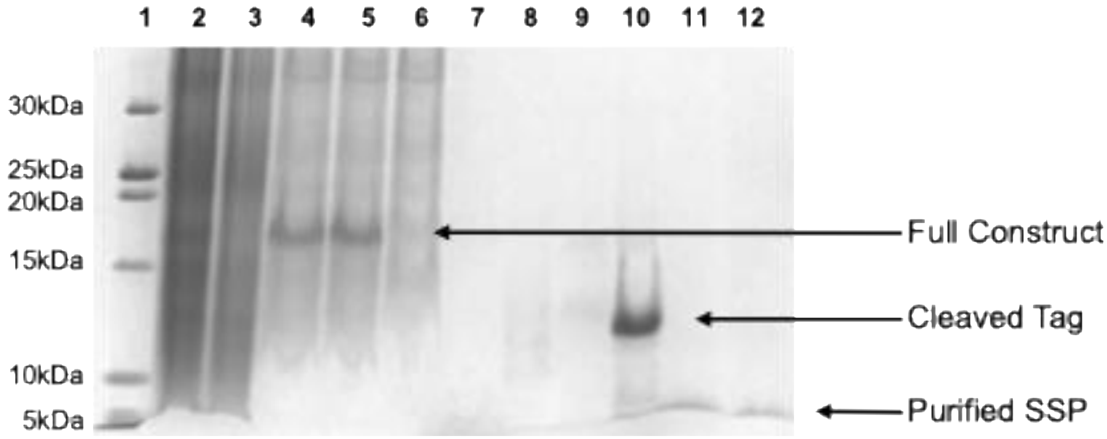

Figure S2. (A) Sequence of LASV SSP construct used in this study. Green=8x His-tag, Blue=TRP-leader tag, Red=thrombin cleavage site, Black=SSP sequence. Underlined residues indicate non-conserved cysteine residues that were mutated to simplify purification. (B) SDS-PAGE gel of LASV SSP purification 1=ladder, 2=pre-centrifugation sucrose buffer, 3=post-centrifugation sucrose buffer, 4=pre-centrifugation binding buffer, 5=post-centrifugation binding buffer, 6=binding flow through, 7=binding buffer wash, 8=wash A, 9=cleavage flow through, 10=wash C, 11=LMPG elution, 12=TCA elution.

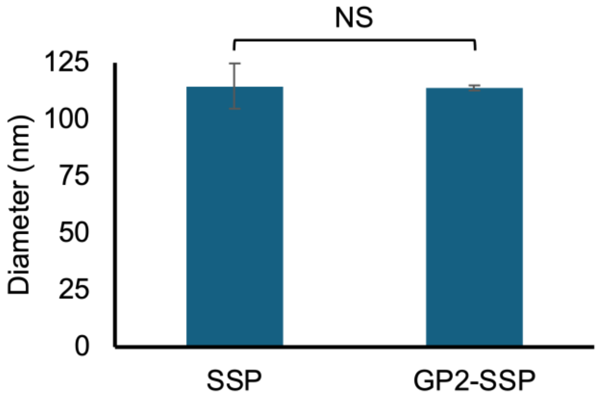

Figure S3. Average size of SSP and GP2-SSP liposomes gathered from DLS was found to be the same. Lipid composition was 75:25 POPC:POPG. Student *t*-Test used to calculate *P*-values; N.S.=Not Significant.

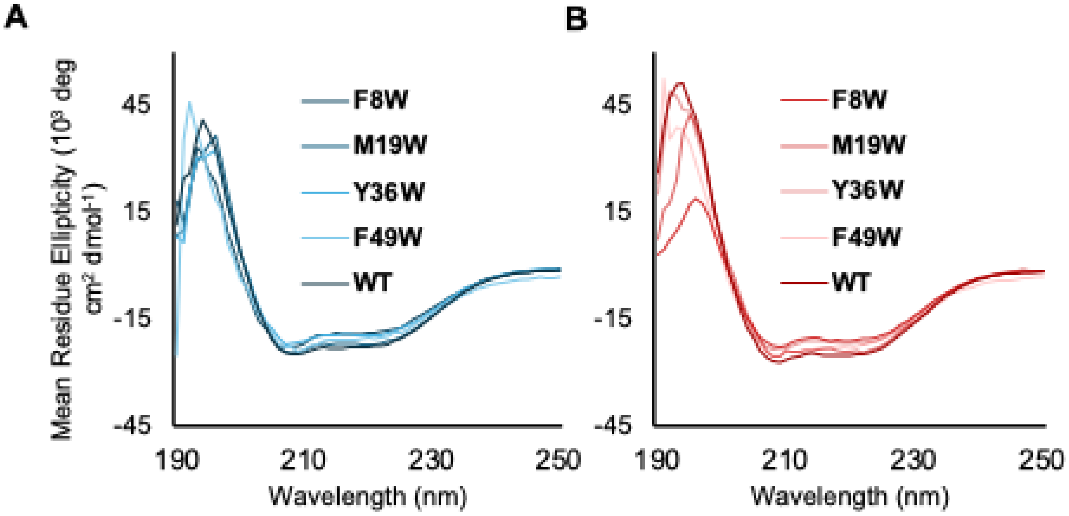

Figure S4. CD spectra of SSP WT and TRP mutants at (A) pH7 and (B) pH4 in LMPG micelles. The Trp mutants do not interfere with the secondary structure of SSP or interfere with its low pH structural change.

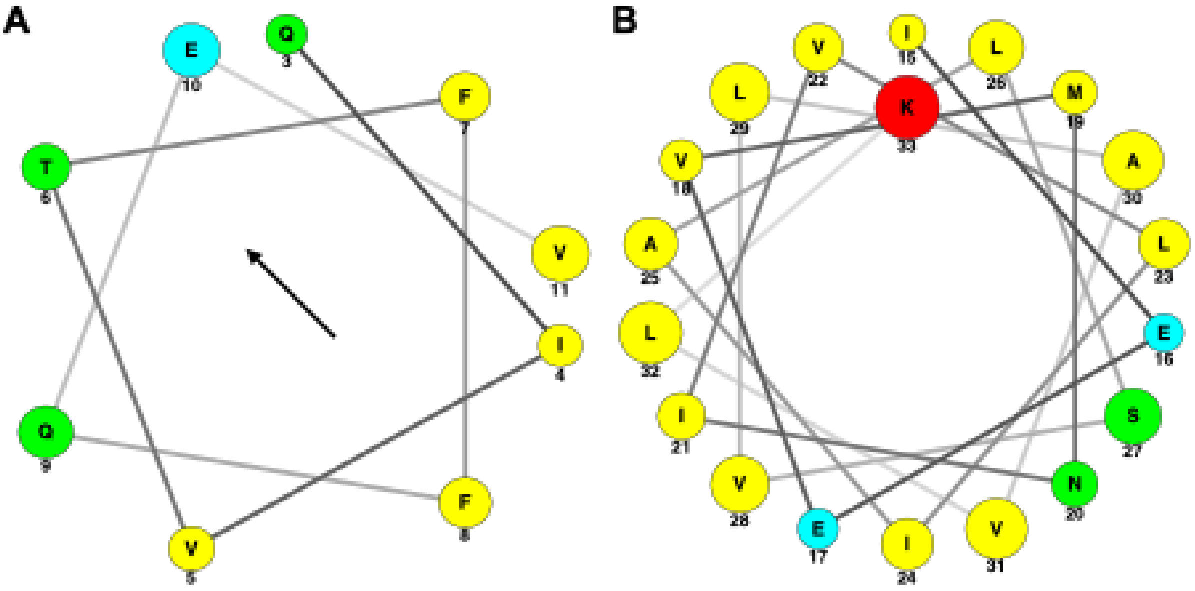

Figure S5. Helical wheel projections of the (A) NTH and (B) TMH. NTH is an amphipathic helix while TMH contains polar-charged residues at its N- and C-termini. Arrow indicates direction of hydrophilic face. Helical Wheels created with NetWheels. Amino acid color scheme goes as follows: Yellow=hydrophobic, Green=polar, Red=acidic, Blue=basic.

Table S1: Compiled fluorescence data of LASV SSP TRP mutants.

		Emission λ_{max} (nm)[a]	Normalized Emission Intensity (RFU)[a]	REES (nm)[b]
**F8W**	*pH7*	333	1	7
	pH6	338	0.99	6
	pH5	332	0.93	12
	pH4	331	0.93	12
**M19W**	*pH7*	329	1	6
	pH6	327	1.05	5
	pH5	326	1.07	6
	pH4	326	1.09	6
**Y36W**	*pH7*	338	1	0
	pH6	338	1.03	1
	pH5	338	1.04	0
	pH4	338	1.02	0
**F49W**	*pH7*	338	1	1
	pH6	339	0.95	1
	pH5	340	1.01	1
	pH4	339	0.93	2

a Excitation=285nm

b Emission=275-290nm
